# Simultaneous Huge Splenic and Mesenteric Hydatid Cyst

**DOI:** 10.1155/2020/7050174

**Published:** 2020-01-29

**Authors:** Mostafa M. Abdelmaksoud, Alaa Jamjoom, Mohamed T. Hafez

**Affiliations:** Surgery Department, East Jeddah General Hospital, Jeddah, Saudi Arabia

## Abstract

Hydatid disease (HD) is caused by Echinococcus granulosus and is endemic in many parts of the world. This parasitic tapeworm can produce cysts in almost every organ of the body, with the liver and lung being the most frequently targeted organs. The spleen and mesentery are unusual locations. We report a case of simultaneous huge splenic and mesenteric hydatid cyst in a 91-year-old male patient. The patient was presented with chronic abdominal pain, increased frequency of defecation, and typical history of animal contact (cattle, sheep, and dogs). After performing imaging studies, he was diagnosed with a simultaneous huge spleen and pelvic mesentery hydatid cyst that was managed surgically by splenectomy, pelvic mesenteric cyst deroofing, and partial cystectomy.

## 1. Introduction

Hydatid disease (HD)—also called hydatidosis, echinococcal disease, or echinococcosis—is a zoonosis caused by tapeworms of the genus Echinococcus; among these organisms, Echinococcus granulosus is the most commonly involved in human infection. HD has a wide geographic distribution and is endemic in many countries—especially in rural regions where agricultural and livestock activities facilitate its transmission. The disease is usually acquired when contaminated water or food that contains the parasitic larvae of echinococci is ingested. Although the liver is the most commonly affected organ and the site of the most well-known associated clinical manifestations, almost any anatomic location can be the host site of the parasitic cysts [1] [2].

## 2. Case Presentation

A 91-year-old male patient presented with one month history of lower abdominal pain associated with increased frequency of defecation as well as dysuria. The patient did not have bloody diarrhea, fever, or any constitutional symptoms. The patient lives in a rural farm with typical history of animal contact (cattle, sheep, and dogs). Abdominal examination was notable for splenomegaly and tender suprapubic fullness. Laboratory tests did not show any abnormalities.

**Figure 1:**
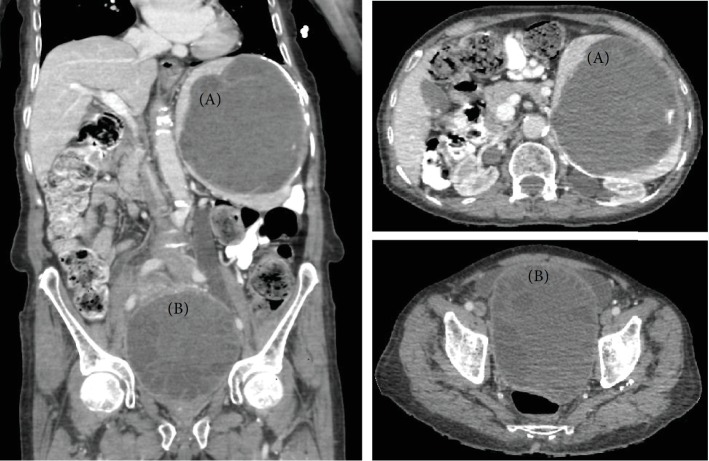
Large hydatid splenic cyst (a), with its size 13.5 × 12.3 × 13.8 cm; and pelvic mesenteric hydatid cyst (b), with its size 14.7 × 11 × 10.8 cm.

The computed tomography (CT) scan of the abdomen and pelvis revealed (Figure 1) a large cystic lesion replacing almost all the spleen with displacement of the stomach and pancreas. A similar huge pelvic cystic lesion is seen at the rectovesical region compressing the urinary bladder and both ureters with bilateral hydronephrosis. The diagnosis was splenic and mesenteric HD.

**Figure 2:**
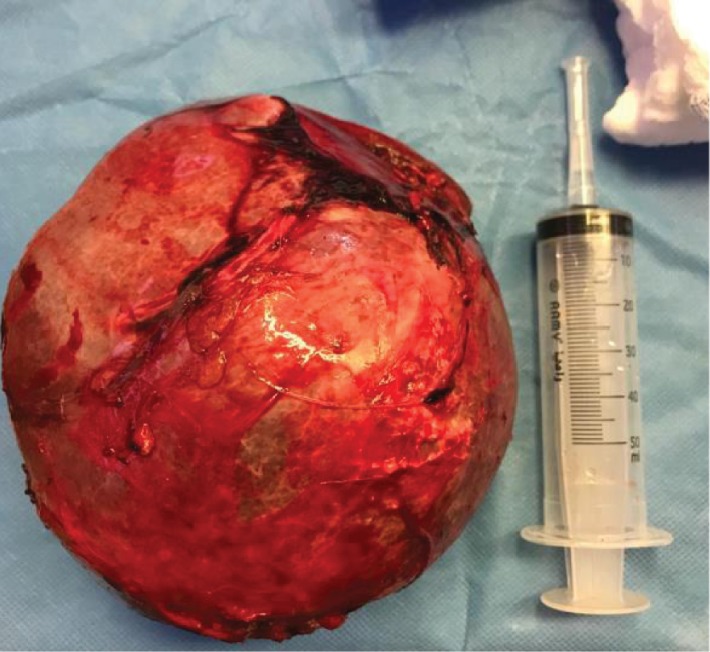
Specimen of the spleen after extraction from abdominal cavity.

**Figure 3:**
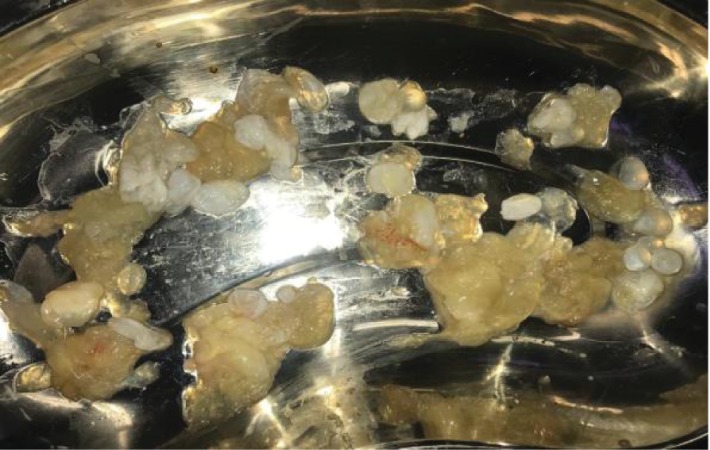
Scolices from the pelvic mesenteric hydatid cyst.

Patient was kept on albendazole 400 mg oral tablet twice per day for 3 weeks before the date of surgery. We started with splenectomy through midline incision (Figure 2). Then proceeding with the pelvis mesenteric cyst that was firmly adherent to the rectum and to the urinary bladder and both ureters. Deroofing of the cyst with partial cystectomy was done. Aspiration of the cyst content (Figure 3) with injection of hypertonic saline was performed without spillage of the contents, followed by excision of an inner germinal layer of the cyst.

Patient had uneventful recovery and was discharged home on the sixth postoperative day to continue on the same dose of albendazole for more 2 weeks.

**Figure 4:**
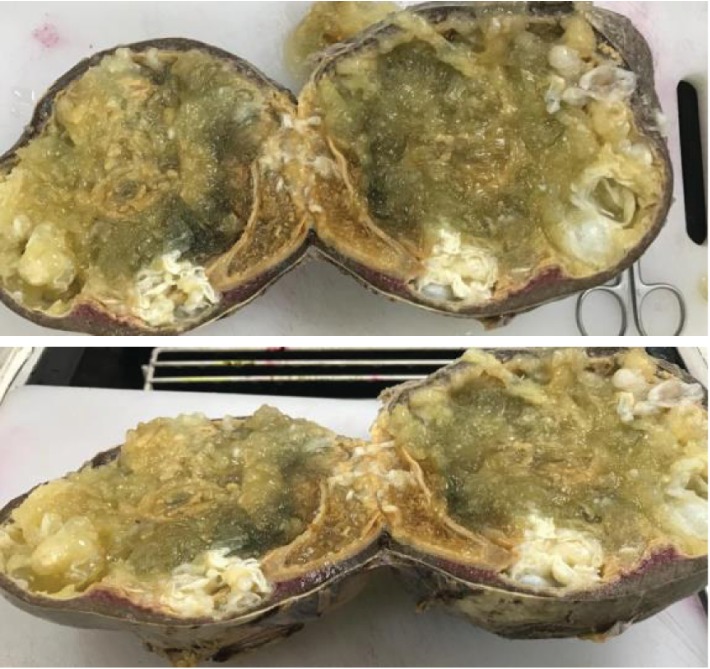
Splenectomy specimen after formalin preservation and cutting at pathology department.

Histopathology examination confirmed the diagnosis of HD of both splenic and mesenteric cysts (Figure 4).

## 3. Discussion

Hydatid disease caused by Echinococcus granulosus exists in endemic cattle- and sheep-raising areas worldwide. Humans are intermediate hosts and become infected by handling infected dogs or other carnivore hosts. Echinococcus usually is asymptomatic, but many cause morbidity and occasional mortality. Infestation by hydatid disease in humans most commonly occur in the liver (55-70%) or the lungs (18-35%) [3–5]. Localization of the hydatid cyst in the spleen is rare, especially when the spleen is the primary and single organ affected by the infection of the parasite Echinococcus. This occurs in about 2% of cases of cystic echinococcosis and occurs when the parasite avoids hepatic and pulmonary filters [6–8].

Splenic hydatid cyst is a diagnostic challenge and treatment challenge [9, 10]. It occurs in the spleen for 0.5-8% of all cystic echinococcosis cases, associated with the appearance of cyst in the liver and other intra-abdominal organs. In about 2% of cases, the spleen is the only organ involved (primary infection) [11] [12]. The disease is endemic in cattle-breeding areas, in South America, Africa, Middle East, South Europe, India, Australia, and Bosnia and Herzegovina [5] [9].

Peritoneal hydatid cyst, either primary or secondary, represents an uncommon but significant manifestation of the disease (approximately 13%). Intraperitoneal hydatid cysts are usually secondary to the rupture (spontaneous, traumatic, or iatrogenic) of a primary hepatic or splenic cyst [13]. Primary peritoneal echinococcosis accounts for 2% of all abdominal hydatidosis [14].

In these cases, surgical procedures can be done (either laparoscopic or open) in addition to drug treatment, or PAIR method (Puncture-Aspiration-Injection-Reaspiration). Within surgical procedures, partial, subtotal, and total splenectomy can be done [10].

In our case, a 91-year-old patient with chronic complains of lower abdominal pain and increased frequency of deification. The patient had a typical history of animal contact being a shepherd in a rural area. The CT scan showed a huge splenic cyst that was almost replacing all splenic tissue and a huge pelvic cyst that was causing bladder and rectum irritation. Our decision was to do splenectomy and removal of the pelvic cyst through midline incision. The patient was kept on Albendazole 400 mg oral tablet twice per day for 3 weeks before the date of surgery.

Splenectomy was performed. Regarding the pelvic mesenteric cyst, it was found intraoperative to be firmly adherent to the surrounding structures including the ureters, urinary bladder, and rectum. To avoid injuries to these structures, deroofing with partial cystectomy was done with excision of the germinal layer. During the procedure, content suction, washing with hypertonic saline, and resuction was maintained carefully without content spillage. The patient had uneventful recovery and was sent home after six days with 2 weeks course on albendazole 400 mg twice a day.

## 4. Conclusion

In endemic areas, hydatid cysts should be considered for the diagnosis of a patient with cystic mass lesions. The radiologic and immunological tests could assist physicians to make the differential diagnosis. Surgery still remains the mainstay in the management of hydatid disease and has to be tailored for each case to avoid unnecessary morbidity.
